# Physical activity behaviors and screen time in young childhood cancer survivors: the Physical Activity in Childhood Cancer Survivors Study

**DOI:** 10.1007/s11764-024-01671-7

**Published:** 2024-09-17

**Authors:** Mari Bratteteig, Corina S. Rueegg, Hanne C. Lie, Lene Thorsen, Elna H. Larsen, Marie H. Larsen, Ingrid K. Torsvik, Miriam Götte, Liisa S. Järvelä, Susi Kriemler, Hanne B. Larsen, Sigmund A. Anderssen, Ellen Ruud, May Grydeland

**Affiliations:** 1 Department of Sports Medicine, Norwegian School of Sport Sciences, Oslo, Norway; 2 Oslo Centre for Biostatistics and Epidemiology, Oslo University Hospital, Oslo, Norway; 3 Epidemiology, Biostatistics and Prevention Institute, University of Zurich, Zurich, Switzerland; 4 Department of Behavioural Medicine, Institute of Basic Medical Sciences, Faculty of Medicine, University of Oslo, Oslo, Norway; 5 National Advisory Unit On Late-Effects After Cancer Treatment, Department of Oncology, Division of Cancer Medicine, Oslo University Hospital, Oslo, Norway; 6 Division of Cancer Medicine, Department of Clinical Service, Oslo University Hospital, Oslo, Norway; 7 Department of Pediatric Hematology and Oncology, Oslo University Hospital, Oslo, Norway; 8 Lovisenberg Diaconal University College, Oslo, Norway; 9 Department of Pediatrics and Adolescent Medicine, Haukeland University Hospital, Bergen, Norway; 10 West German Cancer Center, University Hospital Essen, Essen, Germany; 11 Department of Pediatric and Adolescent Medicine, Turku University Hospital, Turku, Finland; 12 Paavo Nurmi Centre, Unit for Health & Physical Activity, University of Turku, Turku, Finland; 13 Department of Pediatrics and Adolescent Medicine, Copenhagen University Hospital - Rigshospitalet, Copenhagen, Denmark; 14 Faculty of Health Sciences, University of Copenhagen, Copenhagen, Denmark; 15 Institute for Clinical Medicine, University of Oslo, Oslo, Norway; 16 Department of Physical Performance, Norwegian School of Sport Sciences, PB 4014 Ullevaal Stadion, NO-0806 Oslo, Norway

**Keywords:** Cancer survivors, Children and adolescents, Survivorship, Late effects, Physical activity, Sedentary time, Screen time

## Abstract

**Purpose:**

In childhood cancer survivors (CCS), high physical activity (PA) and low sedentary time may reduce risks of late-effects. PA behaviors and screen time, and how they relate to moderate-to-vigorous PA (MVPA) in CCS, are largely unknown. We examined PA behaviors and screen time, and their cross-sectional associations with MVPA.

**Methods:**

CCS from any cancer diagnosis (≥ l year post-treatment), aged 9–16 years at study, were eligible in the international Physical Activity in Childhood Cancer Survivors (PACCS) study. PA behaviors (school transport, intensity-effort in physical education (“PE intensity”), leisure-time PA) and screen time were assessed by self-report, and MVPA by accelerometers (ActiGraph GT3X-BT). Multivariable linear regressions were used to assess associations between PA behaviors and screen time with MVPA.

**Results:**

We included 481 CCS (48% girls, mean age 12.2 years). Passive school transport (prevalence 42%) was associated with 10% lower MVPA/day (*β* = 6.6 min, 95% CI 3.3–10.0), low PE intensity (prevalence 21%) with 16% lower MVPA/day (*β* = 10.2 min, 95% CI 6.0–14.3), and low leisure-time PA (prevalence 34%) with 15% lower MVPA/day (*β* = 9.4 min, 95% CI 1.0–17.7), compared to active school transport, high PE intensity and high leisure-time PA, respectively. High screen time was not associated with MVPA.

**Conclusion:**

Interventions aiming to increase PA behaviors rather than reducing screen time may be more efficient in promoting a healthy lifestyle in CCS through increased MVPA. Encouraging active transport, high PE intensity, and high leisure-time PA seems important in survivorship care.

**Implications for Cancer Survivors:**

Young CCS may benefit from engaging in active transport, high PE intensity, and high leisure-time PA.

**Supplementary Information:**

The online version contains supplementary material available at 10.1007/s11764-024-01671-7.

## Introduction

Childhood cancer survivors (CCS) face higher mortality and morbidity rates than their non-cancer peers [1–3]. A physically active lifestyle may reduce the risk of treatment-induced late-effects among CCS, such as cardiovascular diseases and associated risk factors, osteoporosis, fatigue, depression, and cognitive decline [4–7]. Additionally, sedentary time is associated with frailty in CCS, including exhaustion, low energy expenditure, walking limitations, and weakness [8–10].

We recently showed, using device-measured physical activity (PA), that a cohort of European 9–16-year-old CCS in the Physical Activity in Childhood Cancer Survivors (PACCS) study were less physically active and more sedentary than their healthy peers, and that only one third of these CCS met the World Health Organization’s recommendation of ≥ 60 min of moderate-to-vigorous PA (MVPA) per day [11]. Thus, tailored interventions to increase PA and reduce sedentary time in CCS are warranted. However, we lack knowledge on young CCS’ PA behaviors and screen time, and which activities that are most efficient for increasing MVPA.

Transport to school, physical education (PE), and leisure-time PA represent daily or weekly opportunities for children and adolescents to be physically active [12, 13]. Mode of transportation may depend on several factors, such as age, proximity to school, built environment, culture, and personal/parental preferences [14]. PE is a compulsory class in most countries. However, PE participation and intensity may vary [15, 16]. Leisure-time PA is important for children and adolescents to acquire new, and improve existing skills, as well as for enjoyment, well-being, and interaction with peers [17, 18]. Furthermore, most organized leisure-time PA are of moderate-to-vigorous intensity [13]. Young CCS commonly report to experience PA barriers due to fatigue, and physical and/or cognitive late-effects [15], and having difficulties re-entering organized sports activities after treatment due to a perceived physical ability gap compared to their peers [15, 19].

Sedentary time, and especially screen time, competes with active time among children and adolescents [20]. Moreover, high levels of screen time are independently associated with adverse physical and mental health risk. In CCS, this might be of special concern due to barriers towards PA, and acquired routines of increased screen use from in-hospital education and social interactions via electronic devices during treatment [21].

In sum, there is limited knowledge on PA behaviors, screen time, and their relationship with MVPA in young CCS. In PACCS, we have unique data on both subjective and objective PA, yielding opportunities to study both contexts where CCS are physically active, and how they contribute to volume and intensities of PA. Thus, the aims of this study were (1) to describe PA behaviors in everyday life (transport to school, PE, leisure-time PA) and screen time in young CCS, overall and stratified by socio-demographic, health-, and cancer-related factors, and (2) to explore which of the different PA behaviors and screen time contribute most to the device-measured time spent in MVPA. These results might help tailoring interventions to target behaviors that contribute most to MVPA to reach the recommended healthy levels of MVPA most efficiently.

## Methods

### Study design

The PACCS study is a mixed methods study that has been described in detail elsewhere [22]. The current study comprised cross-sectional data of CCS, from seven hospitals in five European countries (Norway, Germany, Denmark, Finland, and Switzerland).

### Participants

CCS aged 9–16 years with any previous cancer diagnosis and who had completed cancer treatment ≥ l year prior to recruitment were eligible. All eligible CCS at the participating hospitals were invited. Participants were recruited prior to scheduled follow-up visits, and data collection (October 2017 through December 2020) was performed during the visit. Exclusion criteria were cognitive or language limitations that challenged the completion of questionnaires and the wearing of an accelerometer.

### Measures

PA behaviors and leisure screen time were self-reported by the CCS using an electronic questionnaire (Table 1) [23, 24]. The four behavior variables of interest were: school transport (passive, active); PE intensity (low, high); hours of leisure-time PA (low, high); and leisure screen time (low, high). After the enrollment of the first participants, we also added a question regarding the weekly frequency of participation in the following eight types of PA: endurance sports, team/ball sports, esthetics, strength, martial arts, technical sports, extreme sports, and/or other sports.

**Table 1:** Questions used to assess physical activity behaviors and screen time in PACCS

Variable name	Question	Response options and coding	Question origin
School transport (passive, active)	*How do you usually get to school at this time of year?* *How do you usually get from school at this time of year?*	Passive transport was defined as transport by car/motorcycle or bus/tram/subway/train at least one way. Active transport was defined as cycling or walking (or other active) both ways	UngKan (Dalene et al., 2018)^23^
PE intensity (low, high)	*How much do you usually move in PE now?*	Answers were assessed on an ordinal scale from 0 to 7, where 0 was defined as not participating, 1 was “Not much. I never get sweaty or out of breath” and 7 “Very much. I always get sweaty or out of breath”. We categorized 0–4 as low PE intensity, 5–7 as high PE intensity	REPAC (Erdvik et al., 2020)^24^
Leisure-time PA (low, high)	*Besides school-hours, how many hours per week do you engage in sports/exercise that make you feel out of breath or sweat?*	Answers were assessed on an interval scale; 0, 1–2, 3–4, 5–7, 8–10, 11 or more and dichotomized into < 3 h/week (low) and ≥ 3 h/week (high)	UNGHUBRO/FHI Youth Studies 2000–2009—NIPH www.fhi.no
Screen time (low, high)	Asked for weekend and weekdays separately: *On a weekday/weekend-day, how many hours a day, in your spare time (and besides homework), do you usually spend on electronic devices such as TV, PC, tablet or smartphone?*	Answer categories ranged from 0 h/day (coded 0) to ≥ 7 h/day (coded 7). We calculated average screen time ((screen time on weekday*5) + (screen time on weekend-day*2))/7 and dichotomized it into < 3 h/day (low) and ≥ 3 h/day (high)	HBSC (WHO) (Health Behaviour in School-aged Children study) www.hbsc.org
Type and frequency of leisure-time PA (< 1/week, 1/week, > 1/week)	*How often have you been doing the following training activities, in leisure-time, during the last 12 months on average (put a cross next to each activity group)?*	Eight activity-groups were assessed: endurance, team/ball sports, esthetics, strength, martial arts, technical sports, extreme sports, other sports. Four categories of frequency were collapsed into three (never/ < 1 time per week, 1 time per week, several times per week)	UngKan (Dalene et al., 2018)^23^

Abbreviations: *PA* physical activity, *PE* physical education

We assessed MVPA by ActiGraph GT3X + accelerometers (ActiGraph LLC, Pensacola, FL; see criteria in Supplemental Table 1, Supplemental Fig. 1). Participants were instructed to wear the accelerometer on the right hip for seven consecutive days during awake hours, except when swimming and showering. Accelerometer data was downloaded by the ActiLife software (ActiGraph LLC, Pensacola, FL) at each study site, and later uploaded to one secure server for processing in Kinesoft software (version 3.3.80, Loughborough, UK).

**Fig. 1:**
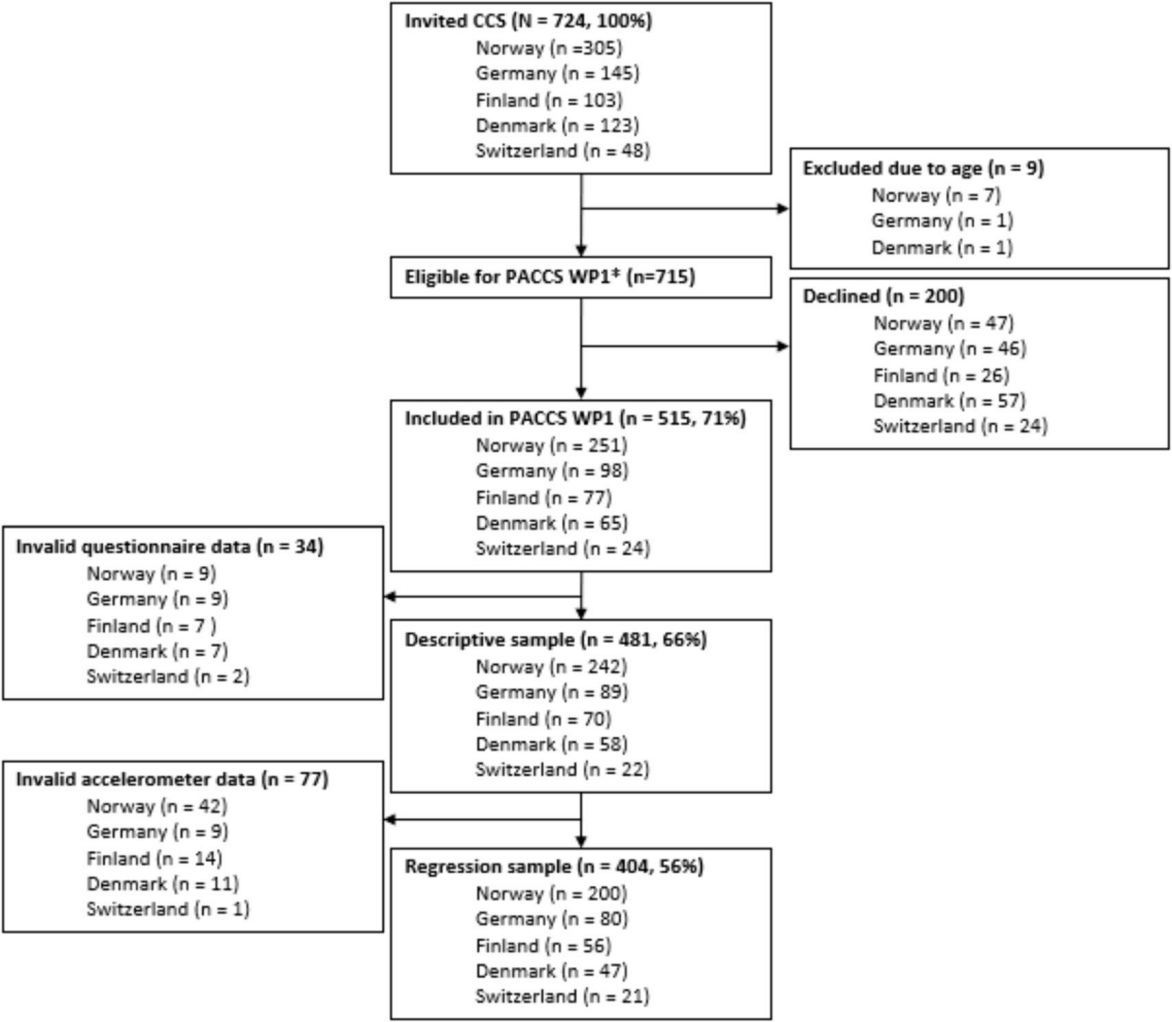
Flowchart of the inclusion process in PACCS work package (WP) 1**.** The asterisk (*) indicates: PACCS comprised of 4 WPs, the current study is based on WP1

We extracted sex and age at study inclusion (continuous or categorized into 9–11 years and 12–16 years) from medical records.

Body mass index (BMI, kg/m^2^) was calculated based on measured height and weight. Values were converted into iso-BMI categories for children and adolescents according to the International Obesity Task Force (IOTF) cut-offs into underweight, normal weight, overweight, and obesity [25]. The IOTF weight status classification are comparable to CDC growth curves and the WHO classification system for weight status in adolescents [26].

Cancer diagnoses were grouped into leukemias, lymphomas, central nervous system (CNS) tumors, solid tumor outside CNS, and sarcomas according to ICCC-3 [27]. Age at diagnosis was categorized into 0–3 years, 4–7 years, and 8–15 years.

Parents self-reported their completed education (categorized into 9–10, 11–13, and > 13 years) in a separate electronic questionnaire (Supplemental Table 2). CCS reported their perceived current health (categorized into bad/not so well and good/very good) [28]. To assess sleep habits, CCS were asked when they usually went to bed and got up on a school day, from which we calculated average hours of sleep on schooldays and categorized it into < 9 h, 9 to < 10 h, and ≥ 10 h [23]. Fatigue was assessed by the PedsQL Multidimensional Fatigue Scale [29]. A total fatigue score was generated and dichotomized into low (score ≥ 70) and high (< 70) fatigue (Supplemental Table 2).

**Table 2:** Characteristics of childhood cancer survivors in PACCS, *n* = 481

	*N* (%) or Mean ± SD
Socio-demographic characteristics	
Country	
Norway	242 (50%)
Denmark	58 (12%)
Finland	70 (15%)
Germany	89 (19%)
Switzerland	22 (5%)
Sex	
Female	229 (48%)
Male	252 (52%)
Age at study (years)	
Mean ± SD	12.2 ± 2.1
9–11 years	218 (45%)
12–16 years	263 (55%)
Parental education	
9–10 years	43 (9%)
11–13 years	164 (34%)
> 13 years	192 (40%)
Missing	82 (17%)
Health-related characteristics	
Iso-BMI	
Underweight	38 (8%)
Normal weight	308 (64%)
Overweight	105 (22%)
Obese	30 (6%)
Self-perceived health	
Bad/Not so well	20 (4%)
Good/very good	461 (96%)
Fatigue (PedsQL score)	
Mean ± SD	75.1 ± 15.1
High	163 (34%)
Low	318 (66%)
Sleep (hours)	
Mean ± SD	9.5 ± 1.0
MVPA (min/day)	
Mean ± SD	63.2 ± 26.0
Cancer-related characteristics	
Diagnostic group	
Leukemia	224 (47%)
Lymphoma	52 (11%)
CNS tumor	77 (16%)
Solid tumor outside CNS	87 (18%)
Sarcoma	41 (9%)
Age at diagnosis (years)	
Mean ± SD	5.1 ± 3.2
0–3 years	265 (55%)
4–7 years	191 (40%)
8–15 years	25 (5%)
Years since diagnosis	
Mean ± SD	7.1 ± 3.3

Abbreviations: *BMI* body mass index, *CNS* central nervous system, *MVPA* moderate-to-vigorous physical activity, *SD* standard deviation

Basic demographic (country, sex, age) and cancer-related variables (diagnosis, age at diagnosis, time since diagnosis, and treatment completion) were available also for non-participants of the current analysis (non-participants; survivors that did not provide consent to participate in the study) and compared between participants and non-participants. The information was extracted from medical records to assess eligibility of the CCS.

### Statistics

All analyses were performed using Stata v17 (StataCorp LLC) and a *P*-value < 0.05 was considered statistically significant. Participant characteristics are presented as mean values ± standard deviations (SDs) or frequencies with proportions.

We presented PA behaviors (school transport, PE intensity, leisure-time PA), and screen time, overall and stratified by socio-demographic (sex, age, parental education), health- (iso-BMI, self-perceived health, fatigue, sleep), and cancer-related factors (diagnostic group and age at diagnosis).

We performed multilevel, multivariable, linear regression models to examine the associations of each PA behavior and screen time with device-measured MVPA. Multivariable models were based on a directed acyclic graph drawn in Dagitty version 3.0 (www.dagitty.net) to identify the minimal set of confounders needed to be included to estimate the total effect of the model: sex, age at study, age at diagnosis, iso-BMI, self-perceived health, sleep, fatigue, country, and season of MVPA assessment (Supplemental Fig. 2). Country was included as a cluster variable. Akaike information criterion (AIC), Bayesian information criterion (BIC), and adjusted *R*-squared were used to investigate which behavior was most strongly related to time spent in MVPA.

**Fig. 2:**
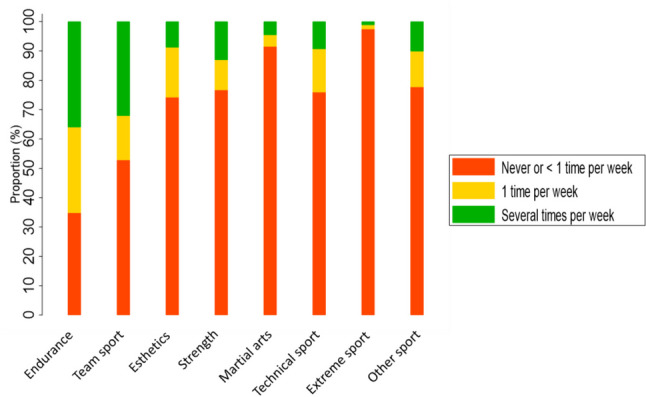
Type and frequency of leisure-time PA in adolescent childhood cancer survivor (*n* = 353). Note: The number of participants in the different leisure-time physical activities ranges from 346 to 353. Categories: Endurance sports (e.g., running, cycling, cross-country skiing, swimming, aerobics); Team/ball sports (e.g., squash, handball, soccer, ice hockey); Esthetic sports (e.g., dance, gymnastics, rhythmic gymnastics); Strength sports (e.g., wrestling, weight training); Martial arts (e.g., judo, karate, taekwondo); Technical sports (e.g., riding, alpine, telemark, athletics, snowboarding, golf, skateboarding, skating); Extreme sports (e.g., river rafting, climbing, paragliding); Other sports (other than the above)

## Results

### Participants

Of 724 invited CCS, 515 participated in the study (71%), 481 (66%) contributed valid questionnaire data (descriptive sample), and 404 (56%) contributed both questionnaire and valid accelerometer data (regression sample; Fig. 1). Half of the participants were recruited from Norway (*n* = 242), and there were some differences between countries in socio-demographic (parental education), health (fatigue), and cancer-related characteristics (diagnostic group, chemotherapy, and surgery; Supplemental Table 3). Participants’ mean age was 12.2 ± 2.1 years, 48% were female, and 64% were categorized as normal weight (Table 2). Near half of the participants had been treated for leukemia (*n* = 224), and the mean age at diagnosis was 5.1 ± 3.2 years. A comparison of participants and non-participants (Supplemental Table 4) showed no differences in basic characteristics.

**Table 3:** Physical activity in school hours: Active transport and physical education intensity stratified by socio-demographic, health- and cancer-related factors, n = 481

	School transport		PE intensity	
	Passive (*n* = 204, 42%)	Active (*n* = 277, 58%)	Low* (*n* = 99, 21%)	High (*n* = 382, 79%)
Country				
Norway	111 (46%)	131 (54%)	51 (21%)	191 (79%)
Denmark	10 (17%)	48 (83%)	12 (21%)	46 (79%)
Finland	24 (34%)	46 (66%)	8 (11%)	62 (89%)
Germany	53 (60%)	36 (40%)	23 (26%)	66 (74%)
Switzerland	6 (27%)	16 (73%)	5 (23%)	17 (77%)
Sex				
Female	92 (40%)	137 (60%)	58 (25%)	171 (75%)
Male	112 (44%)	140 (56%)	41 (16%)	211 (84%)
Age category				
9–11 years	95 (44%)	123 (56%)	43 (20%)	175 (80%)
12–16 years	109 (41%)	154 (59%)	56 (21%)	207 (79%)
Parental education				
9–10 years	26 (60%)	17 (40%)	13 (30%)	30 (70%)
11–13 years	72 (44%)	92 (56%)	27 (16%)	137 (84%)
> 13 years	77 (40%)	115 (60%)	39 (20%)	153 (80%)
Missing	29 (35%)	53 (65%)	20 (24%)	62 (76%)
Iso-BMI				
Underweight	16 (42%)	22 (58%)	5 (13%)	33 (87%)
Normal weight	127 (41%)	181 (59%)	60 (19%)	248 (81%)
Overweight	48 (46%)	57 (54%)	26 (25%)	79 (75%)
Obese	13 (43%)	17 (57%)	8 (27%)	22 (73%)
Self-perceived health				
Bad/not so well	9 (45%)	11 (55%)	11 (55%)	9 (45%)
Good/very good	195 (42%)	266 (58%)	88 (19%)	373 (81%)
Fatigue				
High	77 (47%)	86 (53%)	40 (25%)	123 (75%)
Low	127 (40%)	191 (60%)	59 (19%)	259 (81%)
Sleep				
< 9 h	55 (47%)	62 (53%)	27 (23%)	90 (77%)
9– < 10 h	76 (37%)	128 (63%)	39 (19%)	165 (81%)
≥ 10 h	73 (46%)	87 (54%)	33 (21%)	127 (79%)
Diagnostic group				
Leukemia	90 (40%)	134 (60%)	36 (16%)	188 (84%)
Lymphoma	22 (42%)	30 (58%)	14 (27%)	38 (73%)
CNS tumor	37 (48%)	40 (52%)	24 (31%)	53 (69%)
Solid tumor outside CNS	41 (47%)	46 (53%)	11 (13%)	76 (87%)
Sarcoma	14 (34%)	27 (66%)	14 (34%)	27 (66%)
Age categories at diagnosis				
0–3 years	104 (39%)	161 (61%)	54 (20%)	211 (80%)
4–7 years	88 (46%)	103 (54%)	36 (19%)	155 (81%)
8–15 years	12 (48%)	13 (52%)	9 (36%)	16 (64%)

Abbreviations: *BMI* body mass index, *CNS* central nervous system, *PE* physical education. School transport: Passive: transport by car/motorcycle or bus/tram/subway/train at least one way. Active: cycling or walking (or other active) both ways. PE intensity: Answers were assessed on an ordinal scale from 0 to 7, where 0 was defined as not participating, 1 was “Not much. I never get sweaty or out of breath” and 7 “Very much. I always get sweaty or out of breath”. Low*: categories 0–4, high: categories 5–7. * Including *n* = 12 (2%) not participating in PE. There were no missing values

**Table 4:** Leisure-time physical activity and screen time after school hours, stratified by socio-demographic, health- and cancer-related factors, *n* = 481

	Leisure-time PA		Screen time	
	Low (*n* = 167, 35%)	High (*n* = 314, 65%)	Low (*n* = 234, 49%)	High (*n* = 247, 51%)
Country				
Norway	98 (40%)	144 (60%)	107 (44%)	135 (56%)
Denmark	21 (36%)	37 (64%)	26 (45%)	32 (55%)
Finland	14 (20%)	56 (80%)	31 (44%)	39 (56%)
Germany	29 (33%)	60 (67%)	55 (62%)	34 (38%)
Switzerland	5 (23%)	17 (77%)	15 (68%)	7 (32%)
Sex				
Female	88 (38%)	141 (62%)	113 (49%)	116 (51%)
Male	79 (31%)	173 (69%)	121 (48%)	131 (52%)
Age category				
9–11 years	92 (42%)	126 (58%)	143 (66%)	75 (34%)
12–16 years	75 (29%)	188 (71%)	91 (35%)	172 (65%)
Parental education				
9–10 years	22 (51%)	21 (49%)	18 (42%)	25 (58%)
11–13 years	65 (40%)	99 (60%)	68 (41%)	96 (59%)
> 13 years	47 (24%)	145 (76%)	96 (50%)	96 (50%)
Missing	33 (40%)	49 (60%)	52 (63%)	30 (37%)
Iso-BMI				
Underweight	8 (21%)	30 (79%)	22 (58%)	16 (42%)
Normal weight	102 (33%)	206 (67%)	152 (49%)	156 (51%)
Overweight	39 (37%)	66 (63%)	46 (44%)	59 (56%)
Obese	18 (60%)	12 (40%)	14 (47%)	16 (53%)
Self-perceived health				
Bad/not so well	16 (80%)	4 (20%)	4 (20%)	16 (80%)
Good/very good	151 (33%)	310 (67%)	230 (50%)	231 (50%)
Fatigue				
High	79 (48%)	84 (52%)	62 (38%)	101 (62%)
Low	88 (28%)	230 (72%)	172 (54%)	146 (46%)
Sleep categories				
< 9 h	68 (43%)	92 (57%)	106 (66%)	54 (34%)
9– < 10 h	16 (57%)	12 (43%)	6 (21%)	22 (79%)
≥ 10 h	151 (33%)	302 (67%)	228 (50%)	225 (50%)
Diagnostic group				
Leukemia	73 (33%)	151 (67%)	106 (47%)	118 (53%)
Lymphoma	15 (29%)	37 (71%)	24 (46%)	28 (54%)
CNS tumor	33 (43%)	44 (57%)	47 (61%)	30 (39%)
Solid tumor outside CNS	27 (31%)	60 (69%)	41 (47%)	46 (53%)
Sarcoma	19 (46%)	22 (54%)	16 (39%)	25 (61%)
Age categories at diagnosis				
0–3 years	102 (38%)	163 (62%)	132 (50%)	133 (50%)
4–7 years	60 (31%)	131 (69%)	92 (48%)	99 (52%)
8–15 years	5 (20%)	20 (80%)	10 (40%)	15 (60%)

Abbreviations: *BMI* body mass index, *CNS* central nervous system, *PA* physical activity. Low leisure-time PA: < 3 h/day, high leisure-time PA: ≥ 3 h/day. Low screen time: < 3 h/day, high screen time: ≥ 3 h/day. There were no missing values

### Physical activity behaviors and screen time

Physically active or sedentary behaviors in school hours (school transport and PE intensity) and after school hours (leisure-time PA and screen time) stratified by socio-demographic, health- and cancer-related factors are shown in Tables 3 and 4, respectively. The least favorable behaviors during school hours and leisure-time, in general, were seen in the German and the Norwegian samples. The behaviors were less favorable with increasing iso-BMI, among those who self-reported bad/not so good health (vs. good/very good health), high (vs. low) fatigue, and in survivors of CNS tumor and sarcoma (vs. the other diagnostic groups), except for school transport, where sarcoma survivors less often reported passive transport than the other diagnostic groups. There were no differences between age categories for PA behaviors during school hours, however, during leisure-time, the oldest age category reported both more PA and screen time than the youngest age category.

Participation frequency in leisure-time PA (*n* = 353) was higher in endurance- and team sports compared to the other sport categories (Fig. 2).

### Associations of physical activity behaviors and screen time with moderate-to-vigorous physical activity

The mean time in MVPA was 63.2 ± 26.0 min/day. Table 5 shows the associations of self-reported PA behaviors and screen time with device-measured MVPA. Active vs. passive school transport was associated with 10% more MVPA/day (6.6 min; 95% CI, 3.3, 10.0; *P* = 0.005), and high vs. low PE intensity with 16% more MVPA/day (10.2 min; 95% CI, 6.0, 14.3; *P* = 0.002). Moreover, high vs. low leisure-time PA was associated with 15% higher MVPA/day (9.4 min; 95% CI, 1.0, 17.7; *P* = 0.036), whereas high vs. low screen time was not associated with MVPA (− 1.1 min; 95% CI, − 6.0, 3.7; *P* = 0.552).

**Table 5:** Association of physical activity behaviors and screen time with accelerometer measured MVPA in adolescent childhood cancer survivors (*n* = 404)

Exposure	*β*-coefficient	95% CI	*P*-value	AIC	BIC	*R*-squared
School transport				3644	3660	0.134
Passive	ref					
Active	6.6	3.3, 10.0	0.005			
PE intensity				3640	3656	0.142
Low	ref					
High	10.2	6.0, 14.3	0.002			
Hours of leisure-time PA				3639	3655	0.145
Low	ref					
High	9.4	1.0, 17.7	0.036			
Screen time				3652	3668	0.115
Low	ref					
High	− 1.1	− 6.0, 3.7	0.552			

Abbreviations: *AIC* Akaike information criterion, *BIC* Bayesian information criterion, *CI* confidence interval, *MVPA* moderate-to-vigorous physical activity, *PE* physical education

Notes: Analysis adjusted for sex, age at study, age at diagnosis, iso-BMI, self-perceived health, sleep, fatigue, season, and country (cluster variable)

School transport: Passive: transport by car/motorcycle or bus/tram/subway/train at least one way. Active: cycling or walking (or other active) both ways

PE intensity: Answers were assessed on an ordinal scale from 0 to 7, where 0 was defined as not participating, 1 was “Not much. I never get sweaty or out of breath” and 7 “Very much. I always get sweaty or out of breath”. Low*: categories 0–4, high: categories 5–7. * Including *n* = 12 (2%) not participating in PE

Hours of leisure-time PA: Low: 0–2 h/day, high: ≥ 3 h/day

Screen time: Low: < 3 h/day, high: ≥ 3 h/day

There were no missing values

Of the examined exposures, the model including leisure-time PA yielded the best model fit to examine associations with MVPA (AIC = 3639), followed by PE intensity (AIC = 3640) and school transport (AIC = 3644), and lastly screen time (AIC = 3652). Adjusted *r*^2^-values were similar for the models including school transport, PE intensity, or leisure-time PA, explaining about 13–15% of the variance of MVPA. The model with screen time explained 12% of the variance in MVPA.

## Discussion

There is a large potential for increasing leisure-time PA, intensity in PE, and active transport to and from school in 9–16-year-old European CCS. Moreover, these behaviors seem more influential in increasing MVPA than efforts to reduce screen time.

We found that 42% of CCS passively commuted to school, which is higher compared to peers in respective participating countries [30–34], and far from the aim of < 20% in the Norwegian transport plan [35]. Most survivors (79%) reported high PE intensity, which contrasts with a Swiss study on the general population, showing that only 33% of PE was spent in MVPA [36]. These differences may have methodological or temporal explanations, as the Swiss study assessed PE intensity by accelerometers and was performed in 2005–2006. However, it may also imply that the activity “feels” more intense for CCS. Previous studies have shown that CCS are less physically fit than healthy peers which could lead to a different experience of PA of the same intensity level [37, 38]. Approximately 35% of the CCS reported low leisure-time PA, aligning with findings from the general population. However, participation in team sports was somewhat lower in CCS than in peers (47% among the CCS vs. 68% and 58% among Norwegian 9- and 15-year-olds, respectively) [23], aligning with challenges CCS face in reintegrating into team sports post-cancer [15, 19]. Average leisure screen time in CCS (186 ± 96 min/day), exceeded results from the Swiss Childhood Cancer Survivor Study (*n* = 766, median age at study 12.5 years, 37% leukemia) showing a median screen time of 82 (45–120) min/day [39]. Differences may be explained by parent-report in their study as opposed to self-report in ours. However, this is also higher than the estimated average daily time online (168 min/day) reported in European peers (*n* = 21,964, 9–16 year-olds from 19 European countries, measured in 2017–2019) [40].

Differences between countries regarding the proportion of survivors reporting unfavorable PA behaviors and high screen time may be explained by differences in diagnostic groups, and thus treatment, but also by cultural, geographical- and/or infrastructural issues. For example, two large cohort studies (Steene-Johannessen et al. (2020), *n* =  ~ 15, 000, and Ruiz et al. (2011), *n* =  ~ 2200) found regional differences in accelerometer-assessed PA and sedentary time in European youth, where youth from Central-Northern-European regions were more active and less sedentary than youth from the Southern-European regions [41, 42]. Bann et al. (2019) found large differences between countries in volume and organization of PA and PE in school [43]. Active transport to and from school was more prevalent among children and adolescents when the distance was perceived short and the environments safe and attractive [44]. Aligned with the general population [41], higher iso-BMI seems to correspond with low PE intensity, low leisure-time PA, and high screen time in CCS. Moreover, those reporting worse health and high fatigue reported the same unfavorable PA and screen behaviors.

PA behaviors and screen time varied between diagnostic groups, with survivors of CNS tumor and sarcoma reporting less favorable behaviors. These survivors may struggle more with severe late-effects that interfere with PA, such as impaired motor skills, balance and cognitive dysfunction, neuropathy, and pain compared to other diagnostic groups [45–47], and may require rehabilitation or additional support to increase their PA level, such as a walking companion to school, adjustments in PE, and help to explore “non-traditional” leisure-time PA. How to re-engage in PA after treatment completion has been described as a lacking topic during follow-up care by both young survivors and their parents [15].

Leisure-time PA, followed by PE intensity and school transport, were better indicators of MVPA than screen time. In a Norwegian study from the general population [23], they found that 3–7 and ≥ 8 h/week were associated with 2.2 and 9.2 min higher MVPA/day, respectively. Only 12% of our participants reported ≥ 8 h/week of leisure-time PA, indicating that the majority in the high leisure-time PA group engaged in 3–7 h/week. Hence, leisure-time PA seems to contribute more to CCS’ MVPA than their peers.

Studies in CCS [39] and from the general population [23, 36, 48] have reported that compulsory school sport and active school transport greatly contributed to overall PA hours in adolescents. Reintegrating survivors into PE classes, and providing essential information to schools regarding CCS are crucial. [49]. In a subsample of our study, CCS described the PE-teachers as essential “gate keepers,” as their expectations and attitudes toward the survivors and their limitations affected the survivors’ ability to participate in PE and thus, their motivation for PA [15].

Dalene et al. (2018) found a 2.2 min reduction of MVPA/day per hour increase in screen time [23]. This contrasts our results where we found no associations between screen time and MVPA, suggesting that MVPA is less influenced by screen time in CCS compared to peers. However, in CCS, screen time may be a strategy for energy recovery after PA. As demonstrated, a large proportion of leukemia survivors had both favorable PA behaviors *and* high screen time. In contrast, a large proportion of CNS tumor survivors had unfavorable PA behaviors *but* low screen time.

This study’s major strength is the large, international sample of young CCS from different diagnostic groups, which includes both self-reported data on PA behaviors and screen time, and device-measured MVPA. Self-reported PA is superior in exploring settings and domains, whereas device-measured PA is superior in assessing dose and intensity. On the other hand, self-reported PA is subject to biases, such as recognition-, memory-, and social desirability, and may capture PA behaviors better in some groups than others (e.g., by age, sex, weight status, education) [50], whereas device-measured PA is subject to reactivity bias and limited in measuring water-based activities, activities with little vertical acceleration (e.g., cycling) and stationary activities with high load (e.g., strength training).

We had an overall high study response with no differences between participants and non-participants concerning basic cancer characteristics, supporting the generalizability of our findings. However, the sample was unevenly recruited across countries, which may affect generalizability. Almost all participants reported having good/very good health, which may imply a healthy volunteer bias in our sample, or that CCS experience a response shift (a different reference to what they consider “bad/not so well health” as they have been through a cancer disease) [51]. Lastly, after March 2020, the COVID-pandemic affected the recruitment, data collection, and possibly behaviors of participants.

The cross-sectional study design hinders conclusions on causal relationships of the results. However, the data are important for tailoring future intervention studies on PA behaviors and screen time in young CCS to their likings and preferences.

## Conclusions

CCS represent a population with long-term health risks, and clinicians should recognize the potential for improving PA behaviors, and address this during follow-up care visits. Leisure-time PA, high-intensity PE, and active transport are important contributors to increase MVPA. Parents and PE teachers can also contribute to survivors’ daily MVPA by encouraging these activities. Our results suggest targeting PA behaviors as opposed to screen time holds greater potential for boosting MVPA in young CCS.

## Supplementary Information

Below is the link to the electronic supplementary material.

Supplementary file1 (DOCX 330 KB)

## Data Availability

The data are not publicly available to preserve individuals’ privacy under the European General Data Protection Regulation.

## Abbreviations

AIC: Akaike information criterion
BIC: Bayesian information criterion
BMI: Body mass index
CCS: Childhood cancer survivors
CNS: Central nervous system
cpm: Counts per minute
HSCT: Hematopoietic stem cell transplantation
ICCC-3: International Classification of Childhood Cancer, third edition
MVPA: Moderate-to-vigorous physical activity
PA: Physical activity
PE: Physical education
