# Supplementary material for: Physical activity behaviors and screen time in young childhood cancer survivors: the Physical Activity in Childhood Cancer Survivors Study
Source: J Cancer Surviv. 2024 Sep 17;20(2):554–65. doi: 10.1007/s11764-024-01671-7 (PMC12988952; doi:10.1007/s11764-024-01671-7)
Supplement: Supplementary file 1 — Supplementary file1 (DOCX 330 KB) [file 11764_2024_1671_MOESM1_ESM.docx]

**Supplemental Tables**

**Supplemental Table 1**. Accelerometer settings and processing criteria in the PACCS study

|  | **PACCS criteria** |
| --- | --- |
| **Sampling frequency** | 30 Hertz |
| **Acceleration assessed** | Vertical axis |
| **Epoch length** | 10 seconds |
| **Non-wear time criterion**  **(consecutive zeros:interruption)** | 20:0 minutes |
| **Valid day criterion** | ≥ 480 minutes |
| **Number of valid days to be included in the analyses*** | 1 day |
| **Intensity cut-off points**** | Evenson: MVPA ≥2296 counts per minute |
| **Season of assessment (based on accelerometer start date)** | Spring: March – May, Summer: June - August, Fall: September - November, Winter: December - February |

*We compared total physical activity (daily counts per minute) by the number of valid days, adjusted for multiple comparisons (Bonferroni correction), and found no significant differences in total physical activity by the number of valid days (Supplemental Figure 1). Thus, participants with ≥1 valid day of accelerometer registration were included in our analyses.

** Evenson KR, Mota J. Progress and future directions on physical activity research among youth. J Phys Act Health. Feb 2011;8(2):149-51.

**Supplemental Table 2.** Questions used to assess health-related and socio-demographic data in PACCS

| **Variable** | **Question** | **Response options and coding** | **Question origin** |
| --- | --- | --- | --- |
| **Socio-demographic data** | | | |
| Parental education (self-reported by the parents)* | *What is your longest completed education?* | We collapsed six categories into three: 9-10 years, 11-13 years, and >13 years of education. | Self-made |
| **Health related data** | | | |
| Self-perceived health (bad, good) | *How is your health now/at this time?* | Four categories were collapsed into two (bad/not so well, good/very good). | Young-HUNT  (Holmen et al., 2014)^28^ |
| Duration of sleep on schooldays was assessed by two questions: | *When do you usually go to bed?*  *When do you usually get up on a school day?* | Available categories were:  1. “Before 8:00 p.m.”  (coded as 4 hours)  2. “Between 8-9 p.m.”  (coded as 3.5 hours)  3. “Between 9-10 p.m.”  (coded as 2.5 hours)  4. “Between 10-11 p.m.”  (coded as 1.5 hours)  5. “Between 11-12 p.m.”  (coded as 0.5 hours)  6. “After 12.00 p.m.”  (coded as 0 hours)  Available categories were:  1. “Before 6.30 a.m.”  (coded as 6.5 hours)  2. “Between 6.30-7 a.m.”  (coded as 6.75 hours)  3. “Between 7-7.30 a.m.”  (coded 7.25)  4. “Between 7.30-8 a.m.”  (coded 7.75 hours)  5. “After 8:00 a.m.”  (coded as 8 hours).  Hours before and after midnight were summarized to create a variable for total hours of sleep on schooldays, categorized it into <9 hours, 9 to <10 hours, and ≥10 hours. | Ung-KAN (Dalene et al., 2018)^23^ |
| Fatigue (high, low) | 18 items related to three domains of fatigue (6 items in each domain): general fatigue (e.g., “I feel too tired to do the things that are fun”), sleep/rest fatigue (e.g., “I rest a lot”), and cognitive fatigue (e.g. “I am easily distracted, I find it hard to concentrate”). | Items were rated on a 5-point Likert scale from 0 (= never) to 4 (= almost always). The numbers were reversed (0=100, 1=75, 2=50, 3=25, and 4=0) and a fatigue score generated by averaging all 18 items. Higher scores are interpreted as less fatigue. High fatigue was defined as a total score <70, whereas low fatigue was defined as a total score ≥70. | PedsQL Multidimensional Fatigue Scale, (Varni et al., 2002)^29^ |

* Parental education was self-reported by the parents/guardians in a separate electronic questionnaire. Any one parent per participant responded.

| **Supplemental Table 3.** Comparison of participants by countries in WP1 (N=481) | | | | | | | |
| --- | --- | --- | --- | --- | --- | --- | --- |
|  | **Norway** | | **Denmark** | **Finland** | **Germany** | **Switzerland** | **P-value** |
|  | **(N=242)** | | **(N=58)** | **(N=70)** | **(N=89)** | **(N=22)** |  |
| **Socio-demographic characteristics** | | | | | | | |
| **Sex** |  | |  |  |  |  | 0.803 |
| Female | 116 (48%) | | 28 (48%) | 36 (51%) | 41 (46%) | 8 (36%) |  |
| Male | 126 (52%) | | 30 (52%) | 34 (49%) | 48 (54%) | 14 (64%) |  |
| **Age at study** | 12.2 ± 2.2 | | 12.3 ± 2.1 | 12.1 ± 2.0 | 11.9 ± 1.9 | 12.4 ± 2.1 | 0.734 |
| **Age category** |  | |  |  |  |  | 0.670 |
| 9-11 yrs | 112 (46%) | | 21 (36%) | 32 (46%) | 42 (47%) | 11 (50%) |  |
| 12-16 yrs | 130 (54%) | | 37 (64%) | 38 (54%) | 47 (53%) | 11 (50%) |  |
| **Parental education** | |  |  |  |  |  | **<0.001** |
| Missing | 54 (22%) | | 3 (5%) | 12 (17%) | 12 (14%) | 1 (5%) |  |
| 9-10 years | 12 (5%) | | 2 (3%) | 1 (1%) | 25 (28%) | 3 (14%) |  |
| 11-13 years | 69 (29%) | | 31 (53%) | 21 (30%) | 34 (38%) | 9 (41%) |  |
| >13 years | 107 (44%) | | 22 (38%) | 36 (51%) | 18 (20%) | 9 (41%) |  |
| **Health-related characteristics** | | | | | | | |
| **Iso-BMI** |  | |  |  |  |  | 0.035 |
| Underweight | 16 (7%) | | 4 (7%) | 5 (7%) | 8 (9.0%) | 5 (23%) |  |
| Normal weight | 163 (67%) | | 40 (69%) | 41 (59%) | 47 (53%) | 17 (77%) |  |
| Overweight | 48 (20%) | | 10 (17%) | 20 (29%) | 27 (30%) | 0 (0%) |  |
| Obesity | 15 (6.2%) | | 4 (6.9%) | 4 (5.7%) | 7 (7.9%) | 0 (0%) |  |
| **Self-perceived health** | |  |  |  |  |  | 0.305 |
| Bad/not well | 14 (6%) | | 3 (5%) | 1 (1%) | 2 (2%) | 0 (0.0%) |  |
| Good/very good | 228 (94%) | | 55 (95%) | 69 (99%) | 87 (98%) | 22 (100.0%) |  |
| **Fatigue** |  | |  |  |  |  | **0.039** |
| High | 19 (8%) | | 6 (10%) | 1 (1%) | 1 (1%) | 1 (5%) |  |
| Low | 223 (92%) | | 52 (90%) | 69 (99%) | 88 (99%) | 21 (96%) |  |
| **Sleep (hours)** | 9.6 ± 1.0 | | 9.5 ± 1.0 | 9.4 ± 0.9 | 9.5 ± 0.9 | 9.5 ± 0.9 | 0.497 |
| **Cancer-related characteristics** | | | | | | | |
| **Diagnostic group** |  | |  |  |  |  | **0.002** |
| Leukemia | 116 (48%) | | 27 (47%) | 38 (54%) | 32 (36%) | 11 (50%) |  |
| CNS tumor | 28 (12%) | | 17 (29%) | 10 (14%) | 21 (24%) | 1 (5%) |  |
| Solid tumor  outside CNS | 52 (22%) | | 4 (7%) | 7 (10%) | 22 (25%) | 2 (9%) |  |
| Lymphoma | 28 (12%) | | 4 (7%) | 10 (14%) | 7 (8%) | 3 (14%) |  |
| Sarcoma | 18 (7%) | | 6 (10%) | 5 (7%) | 7 (8%) | 5 (23%) |  |
| **Age at diagnosis** | 5.1 ± 3.2 | | 5.3 ± 3.1 | 4.8 ± 3.1 | 4.9 ± 3.3 | 5.5 ± 3.2 | 0.806 |
| **Age at diagnosis category** | |  |  |  |  |  | 0.693 |
| 0-3 years | 139 (57%) | | 28 (48%) | 40 (57%) | 46 (52%) | 12 (55%) |  |
| 4-7 years | 90 (37%) | | 29 (50%) | 27 (39%) | 37 (42%) | 8 (36%) |  |
| 8-15 years | 13 (5%) | | 1 (2%) | 3 (4%) | 6 (7%) | 2 (9%) |  |
| **Time since**  **diagnosis** | 7.1 ± 3.3 | | 6.9 ± 3.2 | 7.2 ± 3.3 | 7.1 ± 3.7 | 7.0 ± 2.3 | 0.990 |
| **Time since**  **treatment** | 5.5 ± 3.2 | | 5.2 ± 3.4 | 5.8 ± 3.3 | 6.0 ± 3.6 | 5.3 ± 2.3 | 0.616 |
| **Chemotherapy** |  | |  |  |  |  | **<0.001** |
| Yes | 236 (98%) | | 46 (79%) | 65 (93%) | 69 (78%) | 21 (96%) |  |
| No | 6 (3%) | | 12 (21%) | 5 (7%) | 20 (23%) | 1 (5%) |  |
| **Surgery** |  | |  |  |  |  | **0.010** |
| Yes | 80 (33%) | | 24 (41%) | 21 (30%) | 47 (53%) | 7 (32%) |  |
| No | 162 (67%) | | 34 (59%) | 49 (70%) | 42 (47%) | 15 (68%) |  |
| **Radiation** |  | |  |  |  |  | 0.055 |
| Yes | 65 (27%) | | 9 (16%) | 14 (20%) | 16 (18%) | 9 (41%) |  |
| No | 177 (73%) | | 49 (85%) | 56 (80%) | 73 (82%) | 13 (59%) |  |
| Numbers are presented in means ± standard deviations or frequency and percentages. P-values from linear regression for continuous variables and chi-squared test for categorical variables. | | | | | | | |

| **Supplemental Table 4.** Comparison of participants and non-participants* in PACCS (n=714)^a^ | | | |
| --- | --- | --- | --- |
|  | **Non-participants** | **Participants** | **P-value** |
|  | **(N=233)^a^** | **(N=481)** |  |
| **Country** |  |  | <0.001 |
| Norway | 55 (23.6%) | 242 (50.3%) |  |
| Denmark | 64 (27.5%) | 58 (12.1%) |  |
| Finland | 33 (14.2%) | 70 (14.6%) |  |
| Germany | 55 (23.6%) | 89 (18.5%) |  |
| Switzerland | 26 (11.2%) | 22 (4.6%) |  |
| **Sex^b^** |  |  | 0.143 |
| Male | 99 (58.9%) | 252 (52.4%) |  |
| Female | 69 (41.1%) | 229 (47.6%) |  |
| **Age** [years]^c^ | 12.5 ± 2.2 | 12.2 ± 2.1 | 0.113 |
| **Diagnosis** [ICCC-3 main group]^c^ |  |  | 0.307 |
| Leukemia | 60 (39.5%) | 224 (46.6%) |  |
| CNS tumor | 27 (17.8%) | 77 (16.0%) |  |
| Solid tumor outside CNS | 65 (42.8%) | 180 (37.4%) |  |
| **Age at diagnosis** [years]^d^ | 5.2 ± 3.7 | 5.1 ± 3.2 | 0.753 |
| **Time since diagnosis** [years]^d^ | 7.5 ± 3.3 | 7.1 ± 3.3 | 0.277 |
| **Time since treatment completion** [years]^e^ | 5.6 ± 3.3 | 5.6 ± 3.2 | 0.933 |
| Numbers are presented in means ± standard deviations or frequency and percentages. P-values from linear regression for continuous variables and chi-squared test for categorical variables. | | | |

*Non-participants: invited survivors that did not provide consent or questionnaire data, information extracted from medical records during recruitment

Missing:

^a^: Missing info on all characteristics from 1 non-participants

^b^: Sex missing among 65 non-participants

^c^: Age and diagnostic group missing among 81 non-participants

^d^: Age at diagnosis and time since diagnosis missing among 128 non-participants

^e^: Time since treatment completion missing among 154 non-participants and 2 participants

**Supplemental Figures**

| **Valid day(s)** | **n** | **Mean cpm/day** |
| --- | --- | --- |
| 1 | 7 | 490 |
| 2 | 8 | 578 |
| 3 | 11 | 487 |
| 4 | 19 | 431 |
| 5 | 45 | 487 |
| 6 | 88 | 509 |
| 7 | 226 | 479 |


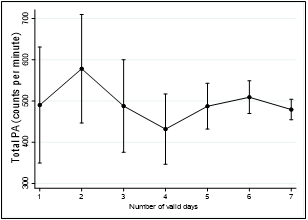


**Supplemental Figure 1:** Participants’ total physical activity according to the number of valid days (n=404). Dots represents mean cpm/day and whiskers represents 95% confidence intervals. P association >0.05. Abbreviations: cpm, counts per minute; n, number.


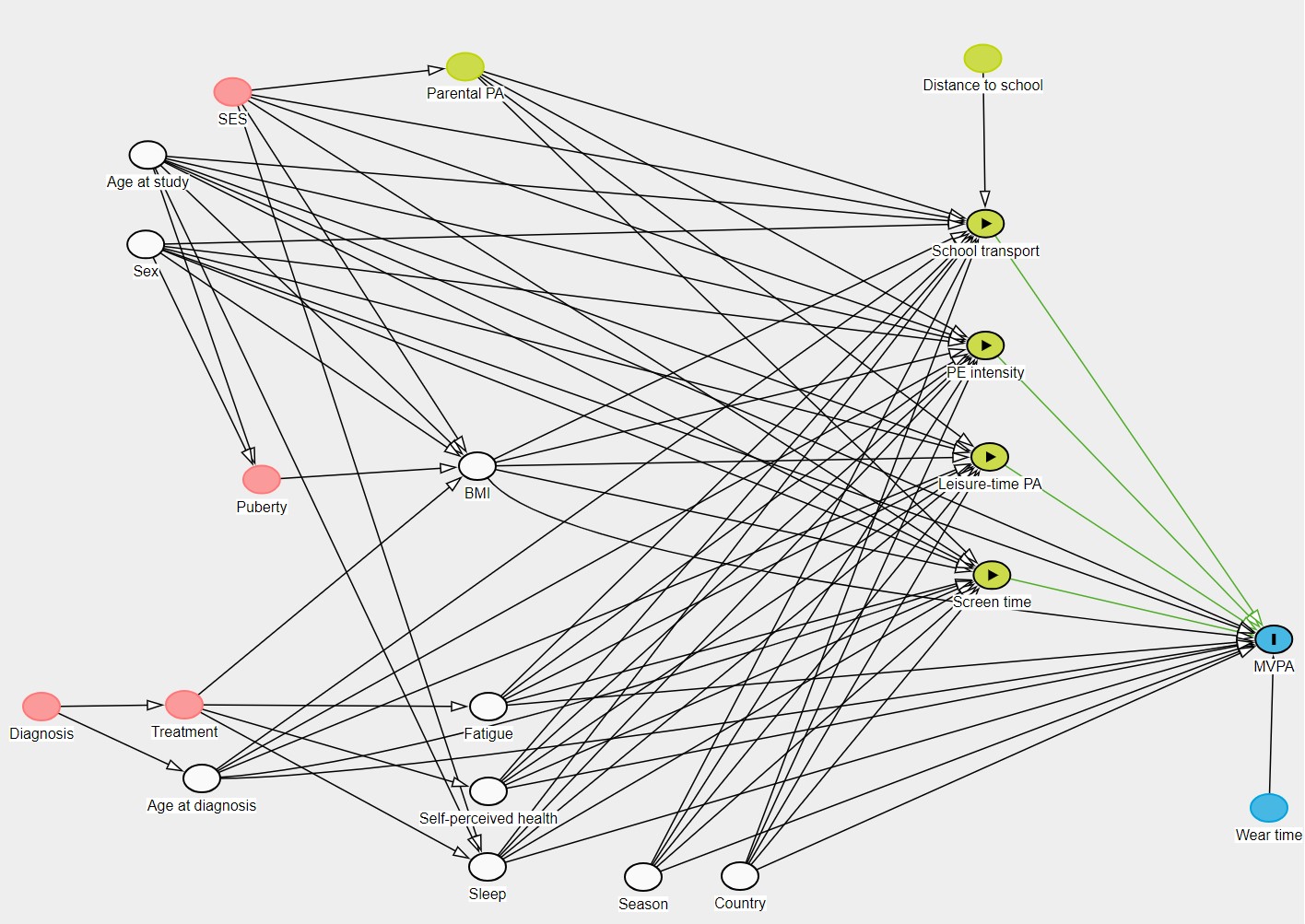
 **Supplemental Figure 2:** Directed acyclic graph used for multivariable regression model specification.
